# Measurement of Emotions Tacting for Empathic Responding (METER): An Example of a Process for Creating an Inclusive Assessment of Emotion Recognition using Validated and Diverse Facial Expression Stimuli

**DOI:** 10.1007/s40617-025-01133-1

**Published:** 2025-12-02

**Authors:** Lydia S. Lindsey, Jaqueline M. Kemp, Sarah M. Richling

**Affiliations:** Department of Psychological Sciences, Auburn University, Auburn, AL 36849 USA

**Keywords:** Assessment, Diversity, Emotion, Tacting, Empathy, Expression, Facial emotion recognition, Inclusion, Inclusive practice, Social skills, Social validity, Validated stimuli

## Abstract

Many social skills, such as empathic responding, social referencing, and facial emotion recognition, require a variety of conditional discriminations under a wide array of stimulus conditions. Proficiency with these responses in the natural environment would involve the ability to identify a variety of emotions across a wide array of faces, genders, ages, ethnicities, and contexts. Using empirically validated stimuli within assessment contexts that represent a wide spectrum of diverse variations across relevant features increases the likelihood of teaching stimulus discriminations necessary for broadly applicable emotion tacting skills. Currently, there is little guidance in behavior analysis on how to conduct a comprehensive assessment of emotions tacting across diverse demographics using empirically validated stimuli. Therefore, this manuscript provides an example process we adopted to create a preliminary assessment of facial emotion recognition that includes empirically validated stimuli representing a multitude of diverse faces, which we named the “Measurement of Emotions Tacting for Empathic Responding” (METER). It is our hope this assessment tutorial will help bring awareness to the importance of identifying appropriate validated and demographically diverse stimuli, the issues that may arise from overlooking the importance of the stimuli we use to assess and teach complex social skills, and to encourage researchers and practitioners to develop inclusive assessments for a variety of social skills using validated and diverse stimuli to aid in developing both targeted and socially valid interventions.

Increased social skills, such as appropriately identifying and responding to others’ emotions and preferences, benefit individuals’ peer interactions (e.g., new peer relationships, increased peer play; Najdowski et al., 2018; Sivaraman, 2017), increase the chances of success in vocational settings (Grob et al., 2019; Morgan & Salzberg, 1992;), and reduce the likelihood of challenging behavior (e.g., Conallen & Reed, 2012; Mann & Karsten, 2020). These social skills are evoked by a variety of discriminative stimuli (SDs) in the natural environment that can vary among many different dimensions, including physical similarities between the stimuli. The first step in many complex social interactions is to observe and label the affect of another person. Thereafter, an appropriate response may involve a conditional discrimination, meaning the response depends on the display of affect by a person in a particular context.

For example, social referencing can be conceptualized as a behavior chain, wherein the learner is confronted by an ambiguous situation, observes the reaction of a communication partner, and engages in an avoidant or approach response depending on the kind of affect displayed (DeQuinzio et al., 2016). The affect functions as an SD (or a contextual stimulus) due to its repeated pairing with consequential events such as extinction, reinforcement, and punishment (DeQuinzio et al., 2016). If there is a deficit in the observing response, including identifying and labeling the affect, then there will likely be a corresponding deficit in the learner’s response to the novel stimuli embedded in the ambiguous situation. These deficits in affect recognition can impact daily functioning, social interactions, and the likelihood of being placed in more restrictive environments (e.g., Kennedy-Hendricks et al., 2016). Furthermore, evidence shows unless directly intervened on, these deficits in social functioning could persist throughout the individual’s lifetime (Gena et al., 1996).

The identification of the facial expression of the communication partner can be described as facial emotion recognition (FER) and is one component of identifying the emotional behavior of others. FER changes throughout the developmental process, as do social experiences, as individuals start to build more peer relationships (Harms et al., 2010). An individual’s age can impact their recognition of others’ facial expressions of emotions. According to developmental theory, FER of the six basic emotions (i.e., anger, disgust, fear, happiness, sadness, and surprise) develops gradually as the individual ages, beginning with happiness and ending with surprise and fear (Herba & Phillips, 2004).

However, age is not the only factor relevant to the development of emotion recognition (e.g., Bänziger et al., 2012; Conley et al., 2018; Herba & Phillips, 2004). Research has shown the importance of multiple factors, including socio-economic status and verbal ability (Herba & Phillips, 2004). Furthermore, the age(s), nationalities, and gender of the expressers (i.e., individuals depicted assessment stimuli) are also key factors in identifying deficits in tacting other’s emotional expressions (e.g., Morningstar et al., 2018). The research on cross-national and multi-ethnic differences in tacting the six basic emotions (i.e., happiness, sadness, anger, surprise, fear, and disgust) found general agreement on the emotions presented, but there were cultural differences in the level of recognition for each emotion (Biehl et al., 1997; Conley et al., 2018; Ekman et al., 1987; Iria et al., 2020). The age, gender, and nationality of the expressers might impact stimulus discrimination for FER. Therefore, it is critical to consider and vary the diversity of stimuli to assess the relevant stimulus control and ensure a robust assessment of the diverse stimuli that could impact responding.

Behavior analytic practitioners are often tasked with teaching social skills that inherently require nuanced consideration of relevant diverse features of the stimuli used for teaching. These social skills may include teaching culturally respectful communication skills, sharing and collaboration, empathy and perspective-taking, understanding and respecting cultural differences, and self-awareness and identity. For example, when teaching culturally respectful communication, clinicians must consider that different cultures have differing contingencies relevant to communication styles and nonvocal behaviors (Mesquita & Frijda, 1992). The clinician must assess the cultural variables present in the natural environment that will shape the contingencies maintaining the appropriate response. The particular stimuli included in the assessment and teaching procedures will influence the likelihood of establishing stimulus control by the relevant diverse features of the stimuli, as well as the likelihood of stimulus generalization and conditional stimulus control.

For instance, a few aspects of the physical properties that may vary are the age, ethnicity, and gender of the person depicted. Research in the field of psychology, more broadly, has shown cross-ethnic mismatch between the learner and the stimuli affects the results of various psychological tasks such as processing and responding to other’s emotional expressions (Conley et al., 2018). Thus, multiple exemplars are recommended for assessments and teaching procedures to promote generalization. To achieve this, clinicians must assess the diversity of the features present in the natural environment to identify the appropriate stimuli to include in the assessment and teaching procedures to actively program for stimulus control and generalization.

Within the behavior analytic skill acquisition literature, there is ubiquitous agreement regarding the need to carefully consider what skills are necessary to be taught in addition to what teaching procedures should be adopted. To this end, formal assessments are often created and implemented to determine: (1) existent skill deficits (e.g., Bradley-Johnson et al., 2008; Gould et al., 2011; Seaver & Bourret, 2014) and (2) the most effective teaching procedure for the learner (e.g., Bradley-Johnson et al., 2008; Carroll et al., 2018; Cengher et al., 2016; Seaver & Bourret, 2014). Individualizing interventions based on the results of these assessments before teaching increases the efficiency of the intervention (Kodak & Halbur, 2021); however, an equally careful consideration of *which stimuli* to include in the assessment and teaching materials is not often described in behavior analytic literature.

Little information appears to be available on what specific stimuli are most frequently used by researchers and practitioners to assess and teach emotion-related skills. It may be that many practitioners use stimuli that are already available within their organization, commercially available stimuli, or stimuli that are easily accessed through an internet search engine. Thus, it is not clear whether stimuli used in such assessments are empirically validated with respect to (a) the development of stimulus control and (b) the generalization and maintenance of the targeted response in the natural environment. Moreover, outside of published research, it is not clear whether stimulus sets used for teaching emotions tacting include a sufficient range of stimulus features that represent different ancestries, ages, or genders. It is also possible stimulus sets could include SDs which might not be as easily generalizable to real life applied contexts such as emojis or cartoon animals. As an example, in some published research behavioral expressions of emotions have been achieved through videos of sock puppets (McHugh et al., 2011) or cartoon characters (Conallen & Reed, 2016). Further, stimuli used to assess emotion recognition might include other complex contextual features (e.g., backgrounds) that block or overshadow stimulus control by the target facial expressions.

Given these considerations, it is crucial for practitioners to consider the nuances of the stimuli selected for assessing and teaching emotion-related skills and other more complex social skills. For example, in early intervention autism clinics, some learners have significant deficits in recognition of emotional expressions and do not readily display generalization of skills across relevant contexts. Therefore, it would be inefficient, and potentially ineffective, to first teach recognition of emotional expression via cartoon stimuli and hope the skill generalizes to actual human faces. As another example, when working with diverse populations, the learner will likely interact with people from various ethnic and racial backgrounds. Primarily using stimuli depicting only people of a single ethnic or racial background may not be appropriate for promoting generalization to the natural environment. However, as previously mentioned, little empirical guidance exists for how to select appropriate stimuli.

To our knowledge, an assessment of emotional expression recognition that includes diverse validated stimuli, differing in terms of ethnicities, genders, and other complex variables, does not exist within behavior analytic literature. Therefore, the purpose of this initiative was to create a practitioner’s guide that demonstrates the process for creating an inclusive assessment including empirically validated diverse stimuli, which we named the Measurement of Emotions Tacting for Empathic Responding (METER). The METER assessment is an example of a preliminary tool designed to assess the recognition of other’s facial expressions of emotions. Through deliberate design, this assessment includes validated stimuli of diverse expressers that we believe can assist with creating learner-specific interventions. The proposed process for creating an inclusive assessment consists of seven steps: (1) identify the target skill; (2) identify existing stimuli; (3) evaluate stimuli; (4) select stimuli; (5) determine assessment stimuli; (6) develop the assessment; and (7) evaluate assessment results. The seven-step process is depicted in Table 1.

**Table 1:** Process for creating an inclusive assessment

#	Step	Brief Description
1	Identify the target skill	Identify the prerequisite skill and select a single modality of the discriminative stimulus to assess
2	Identify existing stimuli	Conduct a thorough search for existing stimuli
3	Evaluate stimuli	Evaluate stimuli according to inclusion criteria and assess validity data
4	Select stimuli	Determine if existing stimuli meet inclusion criteria
5	Determine assessment stimuli	Ensure an adequate number of exemplars are present for the assessment and are different from the teaching stimuli
6	Develop the assessment	Determine the appropriate assessment modality based on the learner’s repertoire
7	Evaluate assessment results	Evaluate the learner’s performance compared to the predetermined performance criteria

## Step 1: Identify the Target Skill

Practitioners may consider targeting social skills that require complex conditional discriminations. However, if specific prerequisite or component skills are deficient, the ability to identify where the deficit lies may be limited in such tasks. A person’s affective behavior involves multiple response modalities (e.g., a person’s facial expressions, gestures, verbal content, and posture) (Gena et al., 1996). There is empirical evidence to support the conceptualization of emotional behavior recognition as a prerequisite skill for empathic responding (e.g., Gena et al., 1996; Sivaraman, 2017).

In the current project, the long-term goal was to design an assessment and individualized intervention plan to teach learners to engage in conditional responding dependent on the affect displayed by another person (i.e., an empathic response). However, if there is a deficit in identifying one of the expresser’s response modalities, there will be a deficit in identifying the combined affective behavior. Sivaraman (2017) assessed the individual’s ability to tact another’s emotions using one modality at a time (i.e., facial expressions and verbal content with social stories) before assessing baseline responding to the combined affective SD. The METER assessment includes static facial expressions in isolation to assess deficits in identifying facial expressions of the six basic emotions and neutral affect using multiple exemplars that more closely match the learner’s natural environment.

## Step 2: Identify Existing Stimuli

Once practitioners identify the targeted skill to assess, the next step is to find existing stimuli depicting the various features of the SDs present in the natural environment. As an initial step, clinicians can conduct a search of the literature for published stimuli that are available for use by the public. This search may include literature outside of the field of behavior analysis, as well as gray literature. Once a stimulus set has been identified, it may be possible to search for related stimulus sets through citations and references.

For example, to determine which stimuli to include in the METER assessment, the first author reviewed existing facial emotion expression databases for their inclusion of certain characteristics, specifically, existing databases including diverse individuals. Because investigators use facial expression research for human and artificial intelligence purposes, the first author evaluated articles and stimulus set databases highly cited and widely utilized in psychological research starting with the NimStem set of facial expressions (Tottenham et al., 2009). The first author then used Google Scholar, PsycINFO, and PubMed® to search for articles citing this stimulus set, related articles, and stimulus sets reviewed and referenced in related articles, specifically looking for diverse sets including validation data.

For inclusion in the analysis for the METER assessment, the database must include static facial expressions presented in isolation on a neutral background without added context. Also, the stimuli must not include additional modalities of emotion expression in combination with the static facial expression (e.g., gesture or verbal phrase). The first author evaluated over 20 different sets according to their validation data and excluded stimulus sets without validation data. This hand search resulted in six databases, as shown in Table 2.

**Table 2:** Descriptive analysis of six existing facial expression stimulus sets

Facial expression stimulus set	Validation assessment characteristics	Overall recognition rate (%)	Racial diversity	Expressers’ age (in years)		Expressers’ sex characteristics			Availability
				Range	*M*^*b*^	Female	Male	Total	
EU-Emotion Stimulus Set — O’Reilly et al. (2016)	Strong	63	Strong	10–70	27	8	9	17	Weak
RADIATE — Conley et al. (2018) https://abcdstudy.org/scientists/abcd-fmri-tasks-and-tools/	Strong	71	Strong	18–30	–	56	53	109	Strong
CEED — Benda and Scherf (2020)	Strong	69	Weak	18–27	20.9	4	4	8	Weak
MPI — Kaulard et al. (2012)	Weak	81	None	20–30	–	10	10	20	Weak
GEMEP-CS – Bänziger et al. (2012)	Weak	47	None	25–57	–	5	5	10	Weak
McGill Face Database – Schmidtmann et al. (2020)	Weak	78	None	23 & 29^a^	–	1	1	2	Weak

Strength of the validation assessment based on number of participants included in assessment: Strong = 100 or more participants, Weak = less than 100 participants. The number of races represented in the set determined the strength of racial diversity: Strong = 3 or more races, Weak = 2 races, and None = 1 race. The complexity of access to the set determined the availability score: Strong = publicly available downloadable file, Weak = request through administrator, None = not available for research or public use. The stimuli for each set can be accessed by contacting the respective corresponding author

^a^ Only 2 expressers used in this stimulus set

^b^ Mean data included for two databases, but researchers did not report mean data for the remaining databases

## Step 3: Evaluate Stimuli

After identifying existing stimulus sets, practitioners must evaluate each set according to the relevant criteria for the target population. The existing stimuli should be evaluated for a variety of factors including but not limited to: (1) the validity of the stimuli, (2) diversity, and (3) how this impacts the skill taught. The stimuli included in the assessment and teaching procedures need to be relevant to the population and should include all the relevant variables impacting stimulus discrimination. Table 3 depicts an example task analysis for evaluating and analyzing the identified stimuli.

**Table 3:**
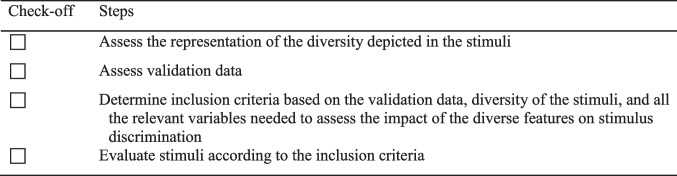
Example task analysis for identifying stimulus criteria and analyzing stimulus relevance for the target population

Practitioners must assess the stimuli identified to ensure that they meet the inclusion criteria (e.g., the relevant diverse variables impacting stimulus discrimination such as age, gender, and ethnicity of the expresser). The assessment should include enough exemplars to fully assess the stimulus discrimination of the relevant variables (see Layng, 2017 for detailed instructions on how to identify defining and nondefining features of a concept). Practitioners can then use these criteria to evaluate the relevance of the stimuli and if the stimuli are diverse enough to adequately assess if these variables impact stimulus discrimination.

After determining the diversity represented within the stimuli, the practitioner can assess the validity of the stimuli identified. One way to assess the validity of the stimuli to the target population is to measure each exemplar’s recognition accuracy by a large number of external observers and evaluate if their accuracy is above chance levels of responding correctly (Bänziger et al., 2012). This method evaluates the social validity and the external validity of the stimuli, or the degree to which the findings generalize to other groups or environments (Kazdin, 1973). This means if an assessment uses these stimuli, the practitioner can infer these results will represent their skills in the natural environment. The validated stimuli also increase the utility of the assessment, as it is now applicable to a wide variety of learners from different backgrounds. Additionally, those scoring the assessment can compare the learner’s recognition accuracy to the mean recognition accuracy for each specific stimulus exemplar. For example, the RADIATE set used in the METER assessment was validated using 662 raters and used two validation metrics to assess the validity of the stimuli: “proportion correct and Cohen’s kappa (Cohen, 1960)” (Conley et al., 2018, p. 1061). One of the benefits of using empirically validated stimuli is that it eliminates the need for practitioners to complete their own validation assessment.

If validation data is unavailable, practitioners can conduct a validation assessment of the stimuli. Another method for conducting a validation assessment is to assess the different individuals present in a learner’s natural environment, such as asking relevant stakeholders to identify the stimuli and collecting interobserver agreement (IOA) data on their responses. This information helps determine the relevance of the stimuli to the target population and increases the likelihood the skill will demonstrate generalization and maintenance under naturally occurring contingencies. However, it is important to assess a variety and sufficient number of stakeholders, as the smaller the number of people assessed, the less likely the practitioner can infer these results will represent the learner’s skills in the natural environment.

Initially, investigators attempted to create a set of stimuli for assessment and teaching by identifying FER stimuli from an internet search, asking graduate students to identify the emotion. Ultimately, the IOA was too low, which led to the search for existing validated stimuli and bypassed the step of conducting a validation assessment.

After determining the validity and representation required for an appropriate assessment of the diverse features potentially impacting stimulus discrimination, the clinician has established their inclusion criteria for evaluating each stimulus set. The clinician can determine the extent to which each identified stimulus set meets the inclusion criteria. After this careful analysis, the clinician can determine which stimulus set to use for the assessment and teaching procedures.

For the METER assessment, the first author evaluated the six databases depicted in Table 2 for their inclusion of specific characteristics. These characteristics included: (a) facial expressions, (b) racial diversity, (c) age range, (d) sex, (e) number of expressers, (f) the availability of the set for use in psychological research, and (g) the characteristics and results of the validation assessment for the entire database. For this assessment, the investigators prioritized identifying as much racial diversity as possible. Sets were classified as “strong” with respect to racial diversity if the stimuli included people of three or more different ancestries, “weak” if the stimuli included two different ancestries, and “none” if the stimuli only included one ancestry. The complexity of access to the set determined the availability score. With regard to accessibility, sets were classified as “strong” if they were publicly available through downloadable files, “weak” if access required a request through an administrator, and “none” if sets were not available for research or public use. The validation was identified as “strong” if the validation assessment included 100 or more participants, “weak” if fewer than 100 participants were used to validate the assessment, and “none” if no validation assessment was done. Table 2 describes these characteristics for each of the databases.

## Step 4: Select Stimuli

Next, practitioners must decide which of the existing stimuli will be included on the basis of their relevance to the target population. If an existing set of stimuli does not meet all the required criteria for inclusion, practitioners must create a set meeting all the previously determined inclusion criteria. Practitioners must also collect validation data for these new stimuli to assess their accuracy and validity using similar procedures to those described previously. Because this is time-intensive, it is recommended to identify a pre-existing, validated stimulus set.

On the basis of the evaluations shown in Table 2, the first author identified one database that included a strong validation assessment (i.e., the database was validated with over 100 participants), acceptable racial diversity (i.e., two or more races were represented), and was available for use with psychological research, the Racially Diverse Affective Expression Face Stimulus Set (RADIATE; Conley et al., 2018). The RADIATE set includes multiple diverse exemplars with 70% or more correct responding (Conley et al., 2018).

## Step 5: Determine Assessment Stimuli

Next, practitioners must determine which stimuli to include in the assessment representing each diverse variable that could influence appropriate stimulus discrimination. To evaluate progress over time, the stimuli included in the assessment must be different than the stimuli included in the teaching procedures. Additionally, the assessment should include a sufficient number of exemplars to represent different combinations of the relevant variables. An ideal assessment would include all defining variables and vary each nondefining variable. The clinician can set aside an additional set of stimuli to use as the generalization probe. Conversely, if feedback is not provided on the learner’s performance, the clinician can repeat the assessment using the same stimuli. Ideally, the assessment should include stimuli with the highest validation score possible. With a higher validation score, there is a higher likelihood of generalization of the skill.

To create the METER assessment, the first author used the eight ethnicity and sex categories designated by the RADIATE set database for each emotion: Asian female, Asian male, Black female, Black male, Hispanic female, Hispanic male, White female, and White male. The names of these categories correspond with the names of categories in the RADIATE set. The assessment contains two exemplars from each of the categories for each of the seven emotions included (i.e., happy, sad, angry, fear, disgust, surprise, and neutral), for a total of 112 exemplars included.

The database contains open and closed mouth versions of each emotion, except surprise and happy exuberant. For the assessment, the first author used whichever open or closed mouth version had the highest validation score for each specific emotion (i.e., the data showed happy open had a higher percentage than happy closed, so investigators included happy open in the assessment). Then, for each emotion, ethnicity, and sex category, the first author selected the top two stimuli with the highest validation percentage for each specific ethnicity/sex combination expressing the particular emotion, since there were multiple actors expressing the same emotion in each of the ethnicity/sex combinations. If more than two stimuli in the same category tied for the highest percentage, investigators used the stimuli not likely to be included for the other emotions. Overall, the assessment includes 73 different actors for the 112 questions (e.g., the assessment includes ten different Asian female actors for the seven different emotions out of 14 opportunities).

The METER assessment includes two exemplars per relevant variable to maintain a brief assessment. In general, the number of stimuli required for different assessments will vary depending on the target population, the targeted skill, and the number of exemplars per relevant variable. For each individual learner, practitioners can choose how many exemplars per relevant variable to include. Environmental conditions and learner-specific differences will influence the length, modality, and implementation procedures of the assessment.

## Step 6: Develop the Assessment

After identifying the assessment stimuli, practitioners must create an assessment using these stimuli, utilizing a format appropriate to the target population. This includes identifying the presentation modality for the assessment, learner-specific considerations (e.g., expressive and receptive language skills, verbal comprehension skills, and barriers to tolerating assessments and cooperating with assessment demands), the response modality appropriate for the given skill, performance criteria, and generalization criteria. An example task analysis for creating the assessment is depicted in Table 4.

**Table 4:**
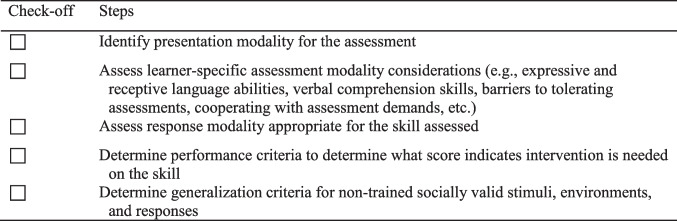
Example task analysis for creating an assessment using the identified diverse validated stimuli

**Fig. 1:**
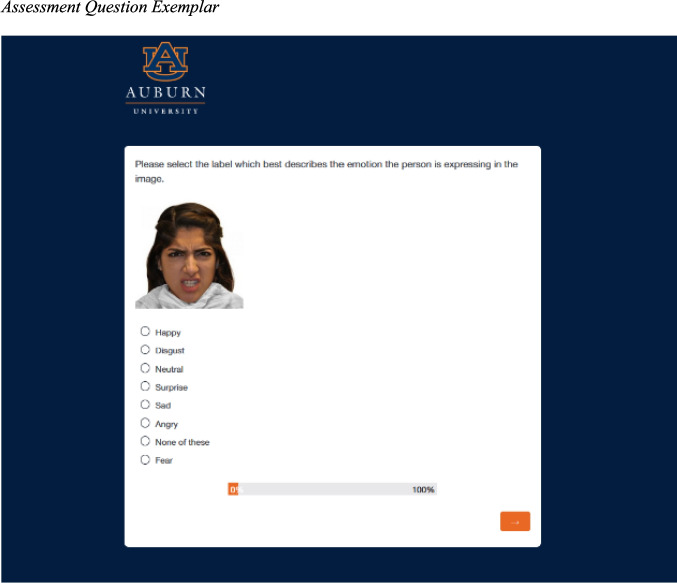
Assessment question exemplar

**Fig. 2:**
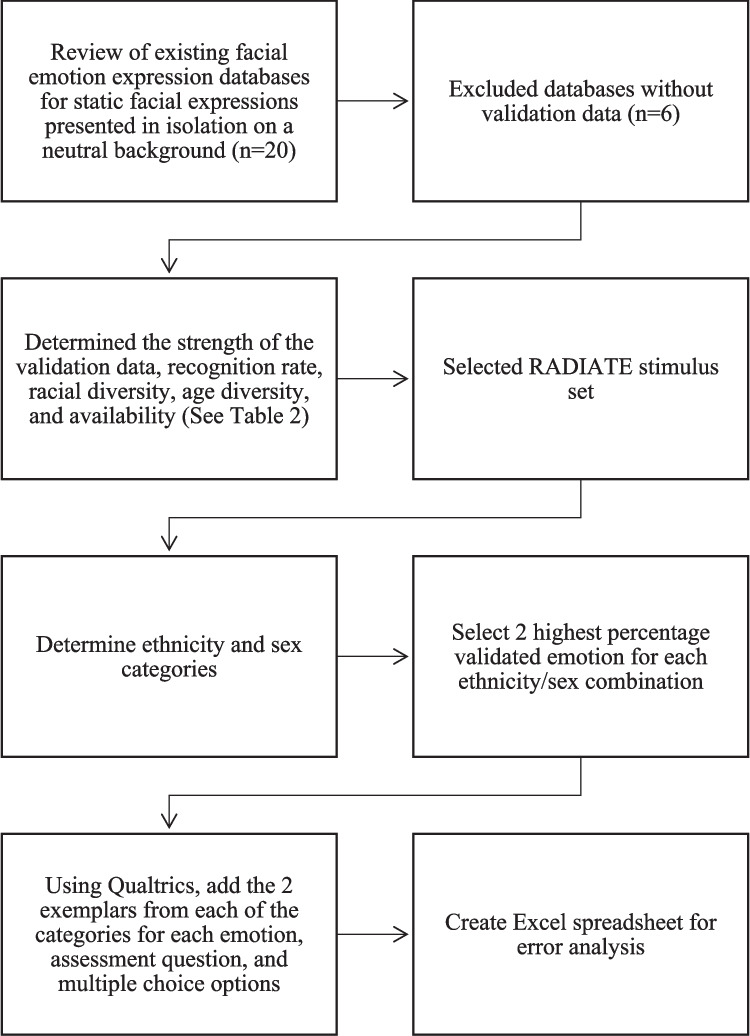
Process for creating the METER assessment

For the METER assessment, each question in the assessment includes a color photo of a facial expression with the instruction to “Please pick the label which best describes the emotion the person is expressing in this image,” as shown in Fig. 1 (specific stimuli used in the assessment and error analysis sheet can be acquired by contacting the corresponding author). The 112 questions were multiple choice questions with eight options: (a) the target emotion, (b) the remaining basic emotions, and (c) none of these. The authors included the “none of these” choice to assist in avoiding artifactually inflated correct answers (Frank & Stennett, 2001). The specific stimulus and all the answer options were present on the screen at the same time, as shown in Fig. 1. There is an unlimited amount of time to answer each question; however, there is not an option to go back and change your response after moving to the next question. Figure 2 illustrates the flow of creating the METER assessment.

Practitioners can modify each assessment format to fit the needs of the learner. The format of the assessment must be appropriate for each learner and practitioners can use the same stimuli in different assessment formats. Practitioners must also determine the length of the assessment and modality of the assessment (e.g., in one session vs. across multiple days, a single assessor vs. multiple assessors, etc.). The METER assessment utilizes multiple choice questions, but practitioners can design an assessment delivered using formats and modalities that have previously been successful with the learner.

## Step 7: Evaluate Assessment Results

After conducting the assessment, practitioners must conduct an analysis of the learner’s responding to identify which skills to target for intervention. Step 6 identified the performance criteria, so practitioners should target for intervention any assessment items which scored below the predetermined criteria using the identified alternative validated stimuli not included in the assessment. Additionally, the learner’s performance can also be assessed against the generalization criteria to determine if the learner’s responding is evoked in the presence of novel stimuli with physical similarities to the previously trained stimuli.

The analysis of the learner’s performance will also be dependent on the modality of the assessment. The performance criteria established the point at which intervention or further assessment was required for the skill. The analysis of the assessment results will determine which of the diverse variables are impacting the learner’s responding and will guide the intervention procedures. Therefore, a thorough error analysis is recommended so that an efficient intervention procedure is implemented to promote generalization to novel socially valid stimuli varying in their representation of diverse features.

**Fig. 3:**
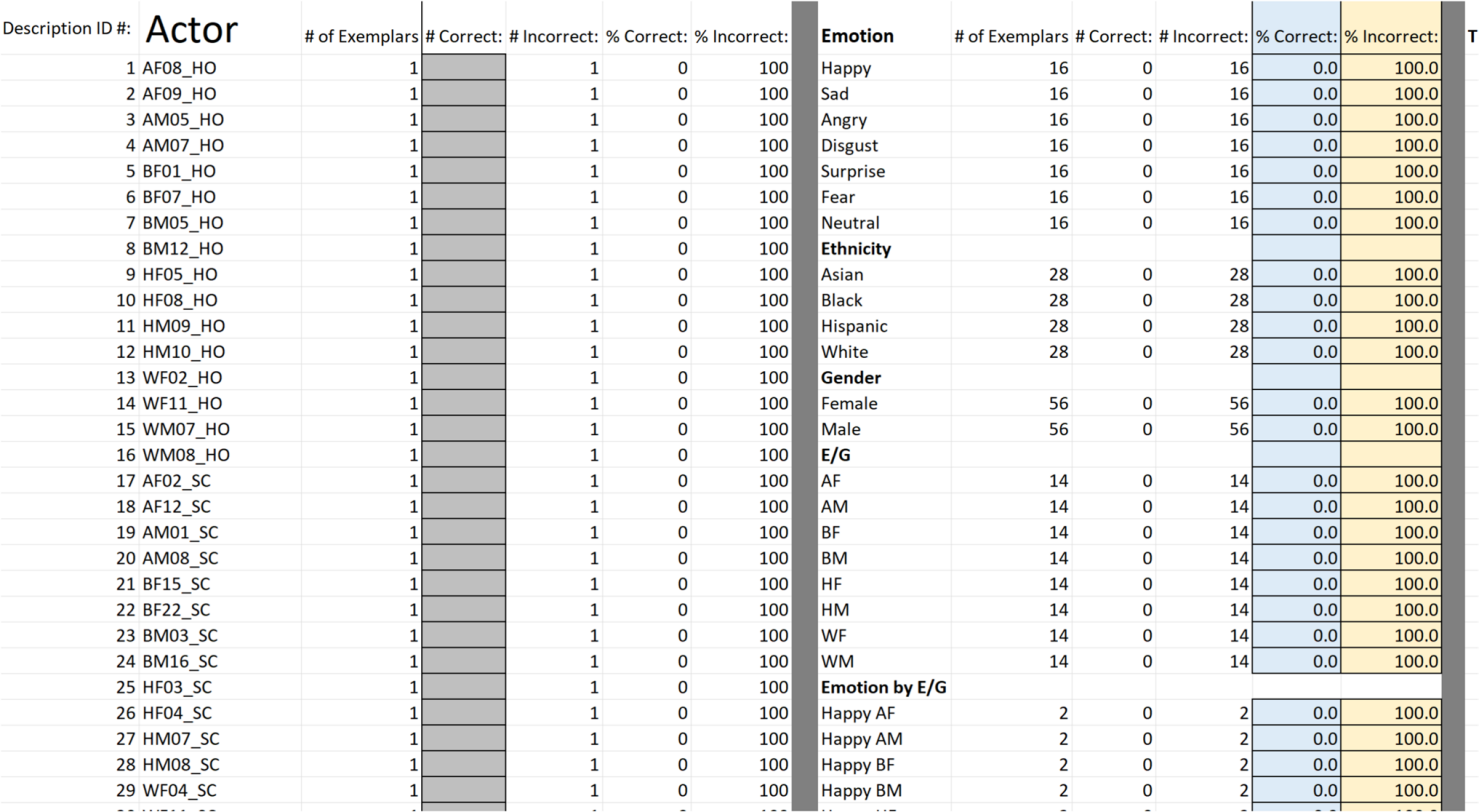
Assessment data entry sheet

**Fig. 4:**
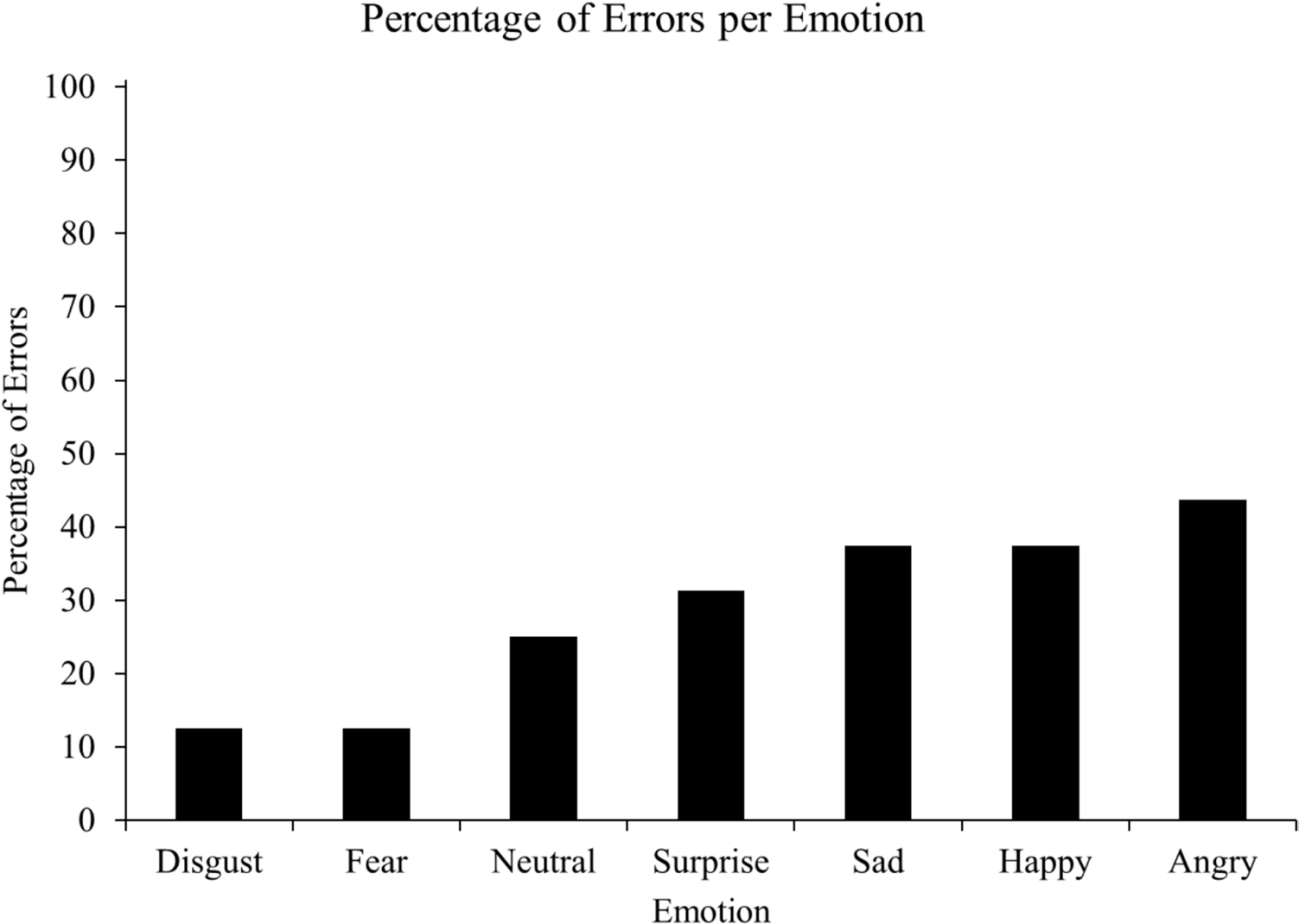
Hypothetical assessment data analysis percentage of errors per emotion exemplar

**Fig. 5:**
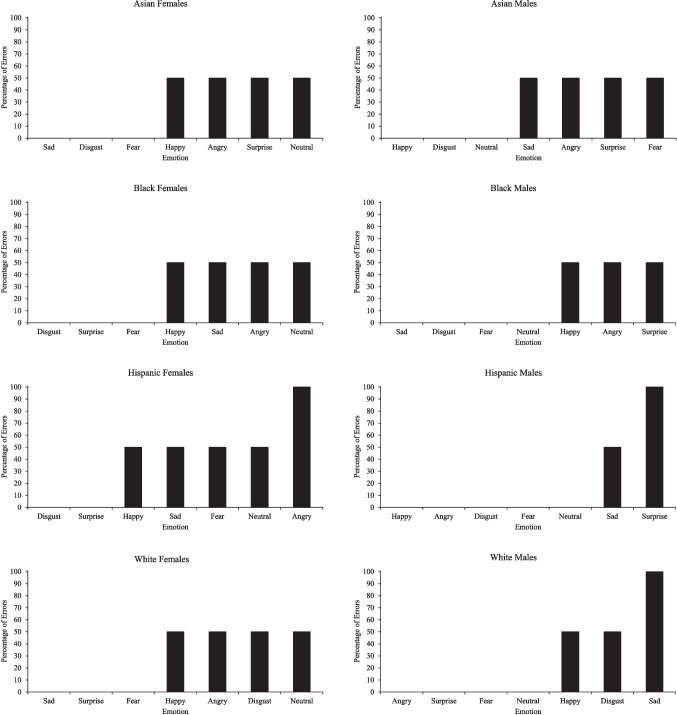
Hypothetical assessment data analysis: percentage of errors per emotion per ethnicity/sex exemplar

For the METER assessment, the investigators uploaded the assessment onto Qualtrics so the assessment could be easily used and distributed within the setting. Once someone completes the 112-question assessment the results can be downloaded as an Excel file. These results can then be entered into a spreadsheet shown in Fig. 3. This spreadsheet is designed to conduct an error analysis to determine which items and stimulus characteristics resulted in a greater number of errors. The spreadsheet calculates the percentage of items scored as incorrect and graphically depicts the results. The spreadsheet includes 19 graphs depicting errors for different variables, including sex, ethnicity, and the basic emotions (see hypothetical data depicted in Fig. 4). The spreadsheet also creates graphs depicting the percentage of incorrect responses for each emotion across the different demographic variables, such as the percentage of errors on Black females depicting happy faces (see hypothetical data depicted in Fig. 5). The results of the assessment and error analysis inform the practitioner on whether teaching is necessary, and if so, for what specific variables.

As shown in the example depicted in Fig. 4, the learner engaged in the highest percentage of errors for anger (35%) and the lowest percentage of errors for disgust and fear (11%). Additionally, the learner engaged in 100% incorrect responding for Hispanic females depicting anger, Hispanic males depicting surprise, and White males depicting sadness. Taken together and depending on the learner’s individualized long-term goals, the practitioner could target Hispanic females depicting angry faces for intervention using the alternative validated stimuli. Once the learner’s responding reaches the mastery criteria for the intervention procedure, the assessment can be conducted again to assess the learner’s responding and potential generalization to diverse expressers of anger that resulted in lower scores (50%) during the first administration (i.e., Asian females, Asian males, Black females, Black males, and White females).

The error analysis allows the practitioner to focus on the specific discriminations that need to be targeted for intervention. This allows for more individualized and efficient teaching procedures and allows practitioners to probe for generalization depending on previously observed error patterns.

## Discussion

One broad purpose of this tutorial was to highlight potential issues with current approaches for selecting stimuli when assessing and teaching socially important skills. In general, there is currently limited emphasis on directing clinicians to carefully and intentionally identify stimuli which will aid in establishing appropriate stimulus control and generalization for social behaviors. This is particularly relevant when teaching exceedingly important and complex skills such as social referencing or empathic responding. A more specific purpose of the current tutorial was to demonstrate how to create an assessment including empirically validated and diverse stimuli, which can be used to identify patterns in error responses. To this end, we introduced an example of such an assessment, which we termed the METER. The METER was designed to assess six basic emotions (i.e., happiness, sadness, anger, surprise, fear, and disgust) and a neutral expression. This can be viewed as a step toward the development of more complex assessments and validation research in the area of emotional behavior within behavior analysis.

The general procedures described in this article can also be viewed as guidelines for clinicians to create inclusive assessments for a variety of skills. For example, when teaching culturally respectful communication, clinicians must consider that different cultures have differing contingencies surrounding communication styles and nonvocal behaviors (Mesquita & Frijda, 1992). The clinician must assess the cultural variables present in the natural environment that will shape the contingencies maintaining the appropriate response. Another example of a program requiring diverse representation is community safety skills. Individuals must respond to a variety of signs, instructions, or authority figures in the natural environment. The defining features present in the stimuli should evoke the appropriate response; however, the nondefining features, such as the font and color of the community sign, may impact responding. Other nondefining features that may vary are the age, gender, and ethnicity of the authority figure presented. These nondefining features are critical to maintaining correct responding in the natural environment.

An ideal comprehensive assessment includes a sufficient number of stimuli that include all the defining features and systematically vary the nondefining features. Further, the set should also include nonexamples (i.e., stimuli that include the same nondefining features but are missing at least one of the defining features necessary for the concept as described by Layng (2017) and Layng (2019). To create a comprehensive assessment, practitioners must evaluate their stimulus sets to assess if the set includes the full range of relevant variable attributes as well as variable nonexamples. However, the number of defining features and nondefining features can increase the number of stimuli required for a comprehensive assessment. Additionally, the increased complexity of the skill may result in an increased number of defining and nondefining features. This could, however, result in a lengthier assessment and may not be appropriate for every learner. Practitioners should therefore carefully consider the stimulus features that are necessary to teach the target discriminations in each case.

Practitioners face potential challenges given the amount of time required to identify existing validated stimuli, evaluate their performance against the inclusion criteria, and create an assessment. However, the time invested is important to appropriately assess the validity of the stimuli used to increase intervention effectiveness and efficiency. As emphasized by Kodak and Halbur (2021), the initial stage of designing an assessment requires significant time, effort, and planning; however, once that stage is complete, the assessment can be utilized across a wide variety of clients for whom the assessment correlates with intervention goals. Practitioners can also work together to identify existing validated stimuli and create a list of resources to use for a variety of skill acquisition programs. Additionally, in some cases, it may be possible to use the same validated stimuli across different skill acquisition programs, different learners, and different assessments.

For the METER assessment, the first author spent approximately five hours researching existing stimuli. The evaluation of the stimuli for the inclusion criteria was completed in approximately five hours. After selecting the existing validated stimuli, the length of time to create the assessment depends on the modality of the assessment and the number of stimuli included in the assessment. The time required to implement the assessment and conduct the error analysis varies and depends on the assessment design. For important prerequisite social skills such as FER, it is recommended to spend the necessary amount of time for careful stimulus selection and assessment of the learner’s performance in baseline across a variety of stimulus dimensions to inform programming.

One particularly useful and notable feature of the METER assessment was the error analysis we developed to help inform specific discriminative stimulus conditions targeted for teaching. Broadly speaking, the purpose of an assessment is to identify high-priority skills intervention as well as skills that are already in the learner’s repertoire. To accomplish this goal, there should be a process for producing a meaningful output from the assessment procedures from which teaching decisions can easily be made. In the case of the METER, this output was the error analysis derived from the assessment results. Conducting an error analysis can lead to efficiency and effectiveness in teaching by identifying: (a) the most prominent skill deficit patterns and (b) behavioral targets that have the greatest potential for producing meaningful outcomes in terms of stimulus control, maintenance, and generalization.

Specifically, our error analysis identified common SDs that produced the greatest number of errors and thereby allowed practitioners to assess where teaching was most needed. For example, a practitioner could identify all exemplar categories with 100% incorrect responding and prioritize those targets. Thereafter, the practitioner could conduct an additional assessment of exemplar categories that previously were associated with lower levels of incorrect responding (e.g., 50% incorrect) to determine if the intervention not only increased the targeted category, but also produced generalization and corresponding increases in accuracy in other categories. Future research should evaluate the most efficient way to teach based on observed error patterns.

If clinicians are interested in extending the METER assessment, this preliminary tool is not without limitations. First, despite the efforts that went into stimulus identification, the assessment is still somewhat limited in its representations of the diverse features found in the natural environment. For example, none of the actors in the assessment were wearing glasses, none reported they were transsexual or nonbinary, and the stimuli do not represent a wide range of ages. These limitations decrease the social and external validity of the stimuli. Additionally, this assessment was created to represent a range of diverse characteristics present in the learner’s cultural environment. However, various cultures differ in their expression and identification of emotions (Mesquita & Frijda, 1992). The METER assessment is based on Western culture’s representations of the basic emotions. Practitioners should consider these cultural differences when designing assessments.

Second, as previously discussed, the assessment included forced choice responding, as opposed to open-ended responding. Most emotional expression tacting assessments, including this assessment, include forced choice responding for more straightforward scoring (Russell, 1994). However, forced choice responding can inflate correct responding and may not be indicative of an individual’s FER abilities under natural circumstances. These limitations reduce the generality of the results. Practitioners can modify their assessments by creating an assessment with open-ended responding and determining coding criteria for responding to aid in scoring the assessment.

Third, the assessment only included static facial expressions to assess one response modality in isolation. Identification of certain facial emotions requires motion (e.g., relief). Dynamic facial expressions include motion, but do not include other response modalities (e.g., posture or verbal content). The identification of the emotion of fear could also increase with the addition of dynamic stimuli, especially since it resulted in the lowest validation score in the RADIATE set (Conley et al., 2018).

A fourth potential limitation of the METER assessment described in this tutorial is that it only includes two exemplars per ethnicity/sex/emotion combination. This limitation was the result of our decision to include only stimuli which met a specific threshold of validity as determined by empirical literature. Unfortunately, despite the adoption of a fairly low criterion for the demonstration of validity, a large majority of the stimuli existing within psychological literature focusing on emotional recognition do not have robust validity for producing consistent responding. Said differently, the stimuli often produce fairly low levels of interobserver reliability with respect to emotions tacting. For example, the RADIATE set includes multiple exemplars per category; however, there are a limited number of stimuli validated above 70% for each category (Conley et al., 2018).

The METER assessment included only stimuli validated at 70% or higher to guard against teaching with unclear or disputable examples of emotions. Given this is an issue in the empirical literature, it is safe to assume that the same will be true for practitioners. Practitioners could also face similar limitations with a lack of existing stimuli for the targeted skill or limited quantities of stimuli available for use. Incidentally, the current assessment was designed as a result of the third author facing this issue when attempting to select appropriate stimuli for teaching empathic responding with adjudicated youth. When collecting relevant stimuli, it was noted that the clinicians on the team labeled the stimuli with differing emotions and were unable to achieve agreement scores above 50%. Therefore, it was questioned to what extent it was reasonable to expect clients to achieve higher scores. Thus, we sought to identify if an empirically validated source of stimuli was available through published research, as described in the current tutorial.

As previously mentioned, the result of this validity criterion resulted in a limited number of stimuli that could be adopted. Specifically, only two exemplars per category were identified. This may not be sufficient to fully assess deficits in FER of the basic emotions or other more complex social skills. Incidentally, the only possible scores for the error analysis of ethnicity/sex/emotion categories are either 0%, 50%, or 100% incorrect responding, depending on whether they incorrectly responded to zero, one, or both exemplars, respectively. Practitioners can determine whether one exemplar is sufficient while taking into consideration that the error analysis will be either 100% or 0% depending on whether the learner responds correctly or incorrectly. Practitioners can also decide to include additional exemplars per category to gain a more comprehensive analysis of the learner’s skills. However, having only two exemplars per category resulted in a relatively brief assessment. When compiling stimulus sets, practitioners could decide to include stimulus sets without validation data, which would potentially result in more sets. If existing stimuli do not exist, practitioners can create stimulus sets by combining exemplars from various sources. However, for both variations, practitioners should plan to conduct their own validation assessments.

Despite these limitations, the development of the METER assessment was intended to be the first step toward the creation of other socially valid assessments and stimulus sets for teaching complex emotional behavior. Applied researchers could aid in this effort by developing and validating more complex stimulus sets, including dynamic stimuli and contextual cues, and determining to what extent acquisition and generalization of skills are reliably achieved. Additionally, future research should include validated stimuli that include additional contextual features to further assess variables impacting stimulus discrimination. For example, crying at a wedding would be tacted as a different emotion than crying at a funeral. This preliminary assessment does not assess a learner’s FER skills in the natural environment but instead provides data on the learner’s ability to identify one key component of the broader skill. Future research should develop assessments for determining a learner’s ability to make conditional discriminations using relevant contextual cues.

The use of dynamic stimuli could also increase the complexity of social skills investigators can include in their assessments and subsequent teaching procedures. For example, similar assessments and stimulus sets could be created for more complex social emotions such as bored, disappointed, and unfriendly which may not be as recognizable in the static form without contextual stimuli or narratives (O’Reilly et al., 2016). However, it is unclear if responding in the presence of more complex stimuli would improve by first teaching in the presence of static stimuli. Future research should develop tools and assess additional emotions which require conditional discriminations and compare the effectiveness of teaching each discrimination in isolation or in combination.

In the ASD research literature, deficits in receptive identification and tacting of facial expressions of emotions are among the most commonly reported (Eack et al., 2015; Harms et al., 2010). For example, research has shown children with ASD have more difficulty distinguishing between nonverbal and verbal content when identifying other’s emotions and tend to rely on verbal content over nonverbal content to identify the emotion displayed (Grossman et al., 2000; Lindner & Rosén, 2006). These deficits may interfere with a learner’s social skills and social interactions across their lifetime (Eack et al., 2015; Kennedy & Adolphs, 2012). Representation is important across a wide variety of social skills, and it is important to consider when selecting stimuli for inclusion in assessments and teaching procedures. However, there is a surprising lack of research identifying appropriate procedures to select these stimuli for assessment and teaching procedures in applied behavior analysis, a field held to be a leader in autism service delivery. Practitioners must recognize the importance of selecting diverse and validated stimulus sets that establish and promote stimulus discrimination across an exhaustive array of stimulus features.
